# Effect of horse sleep behavior on performance in a field-side spatial reversal learning test

**DOI:** 10.1038/s41598-025-34463-9

**Published:** 2026-01-06

**Authors:** Mira Joanna Hämäläinen, Iina Liisa Brotherus, Henna-Kaisa Margareta Wigren, Tuire Eriikka Kaimio, Heli Suomala, Anna-Mari Olbricht, Laura Talvikki Hänninen, Anna Kristina Mykkänen

**Affiliations:** 1 Department of Equine and Small Animal Medicine, Faculty of Veterinary Medicine, University of Helsinki, Helsinki, 00014 Finland; 2 Molecular and Integrative Biosciences Research Programme, Faculty of Biological and Environmental Sciences, University of Helsinki, Helsinki, 00014 Finland; 3 SLEEPWELL Research Program, Faculty of Medicine, University of Helsinki, 00014 Helsinki, Finland; 4 Vahviste, Vantaa, 01690 Finland; 5 Equine Information Centre, Kiuruvesi, 74700 Finland; 6 Department of Production Animal Medicine, Faculty of Veterinary Medicine, University of Helsinki, University of Helsinki, Helsinki, 00014 Finland

**Keywords:** Horse, Behavior, Reversal learning, Sleep, Positive reinforcement, Cognition, Neuroscience, Zoology

## Abstract

Reduced rapid-eye-movement (REM) sleep is associated with impaired learning in many species. We developed a reversal learning test (RLT) suitable for field conditions to explore this association in 16 healthy horses. Nocturnal REM-like sleep behavior was recorded five times for 48 h over a six-week period. The horses performed a target training task followed by an RLT using two objects. When the horses reached a predefined frequency of touching the object, the spatial location was reversed. Mean test parameters were statistically analysed using GENLIN models, longitudinal continuous variables were analysed using linear repeated measures models, and dichotomous repeated measures were analysed using GEE models and Kaplan-Meier method. Altogether 15/16 horses completed RLT by reversing three or more times. Most errors occurred before the second and third reversals. Overall, REM sleep duration (mean ± SE) was 46.1 ± 8.7 min. However, there were ten horses that exhibited REM-like sleep for less than 30 min (10.6 ± 2.2 min, range 0.0–28.0 min), while six horses exhibited REM-like sleep for at least 30 min (42.3 ± 4.8 min, range 36.6–65.8 min). Longer REM sleep was associated with a greater number of reversals (*p* = 0.04), while no relationship was found with error rate. Survival analysis further indicated a significant difference in progression probability between groups, with horses with shorter REM sleep having a 50% chance of progressing after five reversals, compared to six reversals for longer REM sleepers (*p* < 0.05). We present here a method to test horses’ reversal learning ability on site over a single day. We found that short REM-like sleep duration without clinical signs of sleep disturbances in horses was associated with poorer performance and lower perseverance during the RLT.

**Supplementary Information:**

The online version contains supplementary material available at 10.1038/s41598-025-34463-9.

## Introduction

Sleep is essential for learning and memory in all studied species, and insufficient sleep or sleep loss impairs memory consolidation^1,2^ and in waking performance, including executive control, attention, and motivation^3,4^. Both non-REM and REM sleep contribute to learning and memory, with potential task- or memory-specific nuances^5,6^. Equine sleep is polyphasic and primarily occurs at night, with a total daily sleep duration of 2.5 to 5 h including both REM (rapid-eye-movement) and non-REM sleep^7^. Horses frequently fix their patella to allow the contralateral hind limb to rest while standing, which enables them to experience drowsiness and non-REM sleep stages^8–10^. However, muscle atonia—a defining feature of REM sleep^11^ —requires the horse to lie down with a relaxed neck. This posture has been successfully utilized in several studies^7–9,12–14^ and is the only practical behavioral indicator for quantifying REM sleep in horses. Like some other prey animals^15,16^ and those facing hostile environmental conditions^16,17^, also horses possess the evolutionary adaptive ability to reduce total sleep or entirely suppress REM sleep for up to several weeks^12^.

Horses naturally adjust their sleep patterns, and manipulations of husbandry conditions—such as hard and wet bedding and smaller lying areas in both individual pens and group housing—can result in horses spending less time lying down and therefore less time in REM sleep, which can even lead to sleep disturbances^18–21^. However, short-term sleep disturbances have not been shown to affect horse performance in spatial learning^22,23^ or visual attention tasks^23^. On the other hand, studies in other species have shown that both long-term REM sleep deprivation^24,25^ and overall sleep deprivation^23,26,27^ impair cognitive flexibility, leading to reduced performance, lower motivation, and increased cognitive rigidity^27,28^.

Reversal learning tests (RLTs) are used to assess flexibility and the ability to adapt to changing reward conditions, based on the principles of operant and classical conditioning^29,30^. In classical conditioning behavior is triggered by stimulus^31^. Operant conditioning involves the modification of behavior by means of reinforcement, positive or negative, which strengthens the behavior^32^. In practice, during the RLT, the animal is first trained using a food reward, positive reinforcement to establish a new spatial or visual task, and once the animal has learned the rule governing the reward, the rule is then changed^33,34^. Continued observation gauges whether the animal continues to repeat the previously reinforced task, if they fail to adapt to new conditions, or manage to learn the new rule governing the reward^35^. Evidence of learning ability is considered a progressive reduction in the number of errors per reversal^36–39^. The laws of learning are the same regardless of animal species and even fly larvae have the capacity for reverse learning^40^. It is unclear which factors influence reversal learning, but at least the following may hinder successful outcomes: social isolation^41^, sleep deprivation^42^, depression^43^, and brain damage^44,45^. Differences in test design can substantially affect interpretation, as some setups may fail to measure true reversal learning and instead capture other forms of learning or task adaptation.

Earlier reversal learning tests in animals required separate training areas, big environmental changes from the animal’s point of view, and complex constructions like a maze^38,46,47^, a wall^39,48^, or even computer screens^41,42,49^. Habituation is a prerequisite for learning and often time consuming for horses to adapt to dynamic environments^50^. Several factors may affect a horse’s learning ability, highlighting the need for easy-to-use learning tests that can be performed in a familiar environment.

Reversal learning tests have previously been employed in horses to assess learning ability^36,38,46^, trainability^38^, and performance^51^. The very first reversal learning tests in horses were modifications of the Wisconsin General Test Apparatus^33^ combining brightness and spatial cues^36,38^. Two subsequent studies demonstrated that horses perceive spatial location more effectively than visual cues^37,39^. A simpler approach was applied in Warren & Warren’s study, where two horses underwent a series of reversals in the paddock using classical conditioning and positional discrimination with two boxes^36^. These horses solved reversal problems more quickly than the original discrimination tasks and displayed a marked reduction in errors during the serial reversal task^36^. On the other hand, in a maze escape test, horses learned the reversal pattern faster but made more mistakes compared to the initially learned route^38^. When only color was used as a visual cue, no connection was found between reversal learning ability and performance^51^.

The aims of this study were to: (1) create a practical learning test that is easy to use in field conditions; and (2) investigate whether REM sleep behavior explains the differences between horses in the reversal learning test. Our hypothesis was that all horses can learn a simple target training task. Furthermore, we hypothesized that a low amount of REM sleep is linked to impaired cognitive flexibility, and that horses would therefore be able to successfully perform the reversal learning test.

## Materials and methods

We confirm that the procedures complied with national and EU legislation. The research was conducted in accordance with the relevant guidelines and regulations of the Finnish National Board on Research Integrity (https://tenk.fi/en*).* All study procedures were approved by the Research Ethics Committee for Animal Research of the University of Helsinki (license number 1/2023). Prior to participation in the learning test or involvement in the test development, the horse owners provided their informed written consent for the use of their horses’ test results in research. The reporting of results follows the recommendations of the ARRIVE guidelines (https://arriveguidelines.org*).* Informed consent was obtained from each subject or their legal guardian for the publication of identifying images or videos in an open-access publication.

The study was carried out at Hingunniemi YSAO in Finland in November 2022, utilizing horses from the school’s riding program. YSAO, the owner of the horses, granted permission for their use in the research.

### Animals

Sixteen riding school horses (mean ± SE; age 15 ± 1 years; height 160 ± 1 cm) both geldings (14) and mares (2) participated in the test. Horses were Warmbloods (12) and Finnhorses (3). All horses were examined by a veterinarian prior to testing and were deemed healthy. The horses participated simultaneously in an unrelated crossover study investigating different bedding depths of either 5–15 cm of peat. After an initial three weeks, the bedding conditions were switched between the groups to complete the crossover design.

The horses were kept in individual pens measuring 9 m² with visual contact to other horses and spent 8 h each day in a paddock, where they had the opportunity for tactile contact with another horse, either over the fence or in shared paddocks. The horses worked in the riding school 1–2 h a day during the morning and/or evening. Weekends were usually free of work. The horses were fed with concentrates in their own pens at 6 AM, after which they went out to pasture. Hay was offered four times a day, twice outside at 7 AM and at 10:30 AM, and twice inside the individual boxes at 4 PM and 8 PM. The amount of hay was estimated based on the horse’s body condition, approximately 2 to 3 kg at a time. The horses had been living on the premises for at least 9 months and had established paddock mates.

The time spent for the complete test procedure was approximately 1 h with a minimum break of 15 min and a maximum of 2 days between preschooling phase and reversal learning test (RLT). All horses were preschooled and tested over five days mainly during daylight hours during their paddock time.

### The test area

**Fig. 1:**
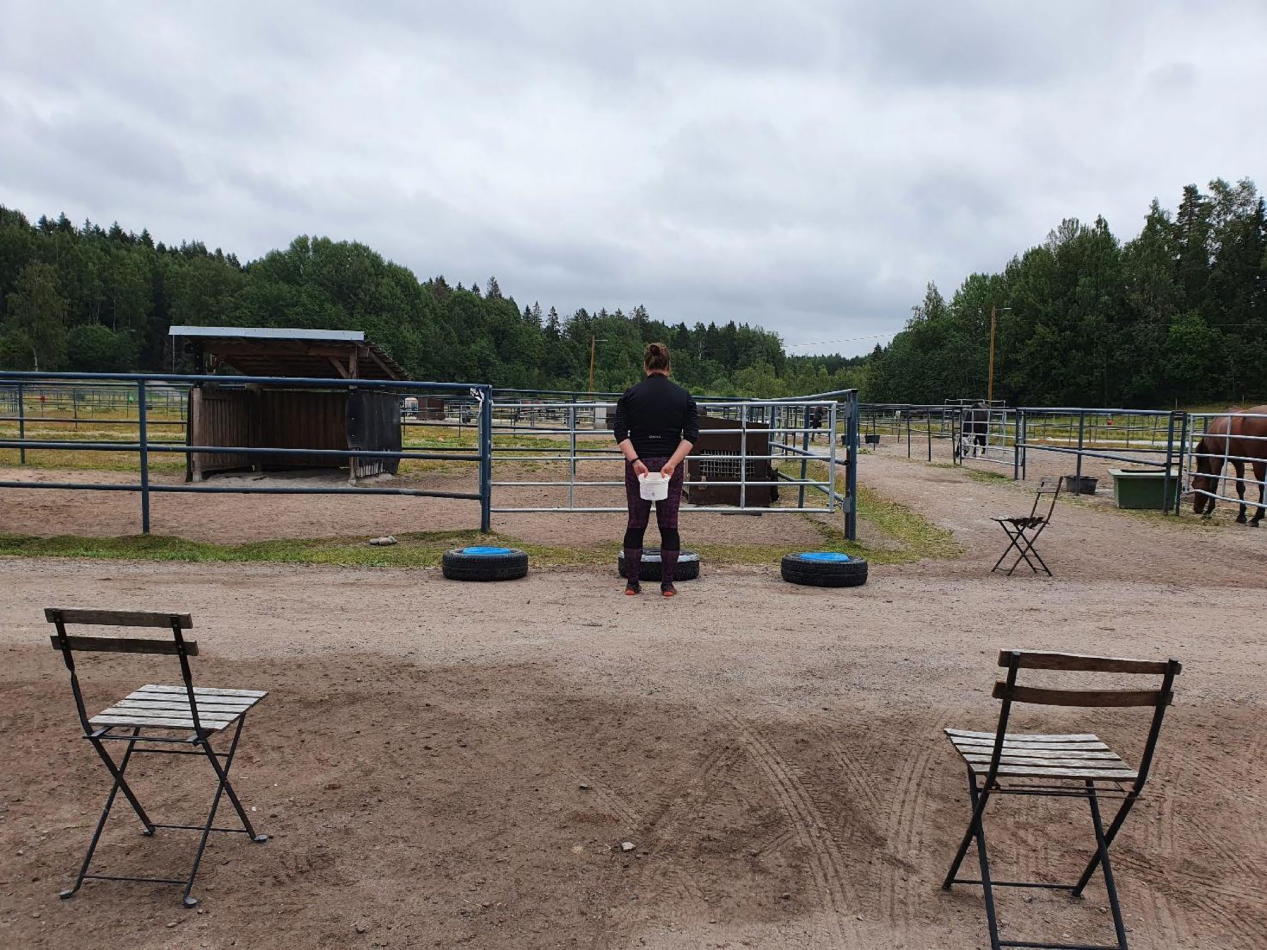
Test area for reversal learning test for horses.

All testing was conducted in an area measuring 3 m wide by the fence, inside horses’ own outside paddocks with the ability to see a familiar horse in the neighboring paddock (Fig. 1). Two to three people were present during study sessions: Experimenter 1, located in the middle of the training area outside the fence, clicked and rewarded the horse and handled the objects. Experimenter 2 was located randomly on the side of the training area, behind Experimenter 1. Experimenter 2 counted and recorded the correct responses reported by Experimenter 1 and informed Experimenter 1 when to reverse sides during the RLT. Experimenter 1 did not know ahead when the reverse was coming. Experimenter 3, an experienced animal trainer (TK), observed the work and body language of the other experimenters in relation to the ‘Clever Hans’ phenomenon^52^ and reminded them about factors such as body alignment and gaze direction^53^. The aim was to prevent the unintentional and repeated cues given to the horse. Experimenter 3 was present during the testing of the first 6 out of 16 horses.

### Objects

**Fig. 2:**
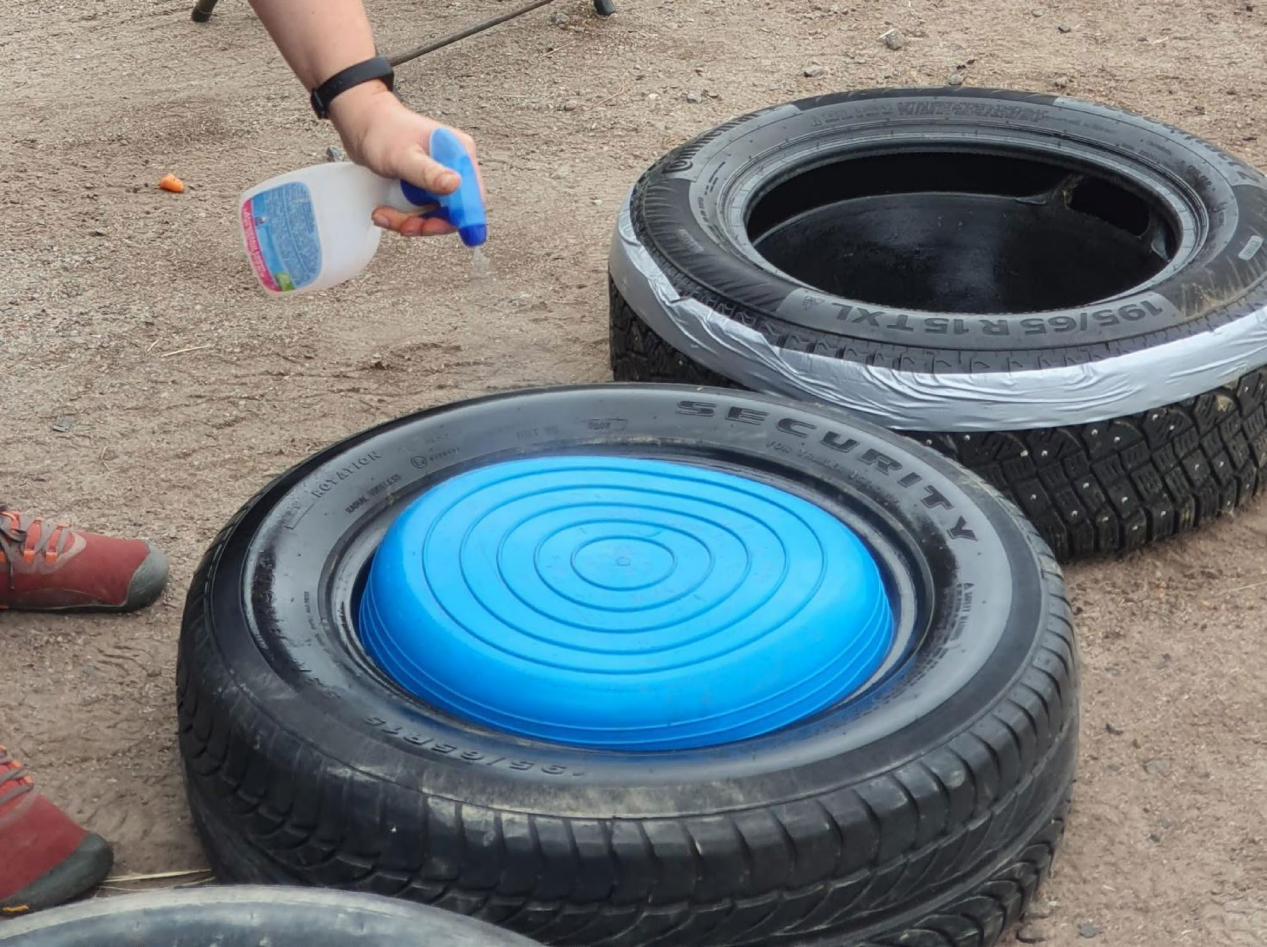
Disinfection of the object and feed bucket used in the reversal learning test for horses.

Car tires without rims were placed on the ground^54^ to hold two identical objects (1 and 2) and one feed bucket in place. In the test area, three car tires were arranged in a row, spaced 90 cm (3 feet) apart. If the horse was smaller than 140 cm in height at the withers the objects were separated by 60 cm. Objects were upside down, blue^55^, flexible, shallow plastic buckets (Gorilla tubs) inside the outermost car tires. The feed bucket for dispensing treats was positioned in the middle: a black rubber feed skip fixed inside the tire with a stripe of duct tape. A horizontal stripe in the middle of the tire enhanced the visual distinction between the feed bucket and the objects, as horses are more attentive to lines and curves than to shapes^56^. These items were tested in pilot tests to be safe, visible, sufficiently heavy, durable for horses, and easy to clean (Fig. 2). Both objects were handled and cleaned similarly during preschooling to ensure a uniform smell and prevent horses from using olfactory cues for discrimination, as scent differences can arise if food is present in the bucket or behind the hatch^36,38,39^. For both the preschooling phase and for the actual RLT, 1 kg of carrots was reserved for each horse in slices of approx. 8 g. Health issues were not detected with this volume of carrots.

### Preschooling phase

The preschooling phase aimed to ensure that all horses were at the same level of learning and became familiar with the objects and positive reinforcement training method. First of all the horse was free in its own paddock with no hay or other food. If a horse normally shared a paddock with another horse, the companion horse was moved to an adjacent empty paddock for the duration of the test. Training sessions were scheduled after feeding and one hour before the next hay feeding time to reduce anticipatory hunger. Before starting the training, we ensured that the horses were fed according to their normal routine (as described above) and had one hour to finish their ration of hay. To ensure that the horses felt safe and calm enough to focus on training, each horse was observed for signs of relaxation, such as ears in a neutral position while monitoring the environment, a soft and relaxed muzzle and lips, a head and neck carried mostly at or below wither height, a relaxed posture, standing square or resting a hind leg, and a willingness to approach unfamiliar people calmly. Stress was identified using an ethogram of established indicators^57,58^, including increased locomotion signs such as bolting, rearing, bucking, or attempts to kick, persistent pawing, vocalization, or startle responses. The same observational checklist was applied to all individuals prior to each session. If the horse was nervous for some reason, we removed distractions and arranged known companion horses nearby and if needed, training was retried again later at another time. Horses displaying persistent stress or pain signals, aggressive responses, or refusal to engage were to be removed from the study; however, no horses met these exclusion criteria.

In the preschooling phase, the horse learns the principle of target work: the horse reaches out toward the blue target with its muzzle, receives an audible signal for correctly performing the task, and a reward is dropped into the feed bucket. Please see instructions on performing all phases of the test in Supplementary Fig. 1. First all the tires (objects and the feed bucket) were outside the paddock and Experimenter 1 dropped a treat to the feed bucket to ensure the horse was unafraid of it. If the horse was uncomfortable, Experimenter 1 continued dropping treats with a shortening distance between the feed bucket and the horse until the horse was habituated and the feed bucket was in its place inside the fence. Just before dropping the treat Experimenter 1 said a secondary reinforcer, a short word that means nothing to the horse ahead, to mark the treat is coming. Different words were used for horses if there was a risk that they might have heard each other’s words. When Object 1 was first introduced, a treat was placed on top of it to non-intrusively lure the horse to touch the object. After the horse ate the treat, it was immediately rewarded with another treat in the feed bucket when it sniffed the object again.

**Fig. 3:**
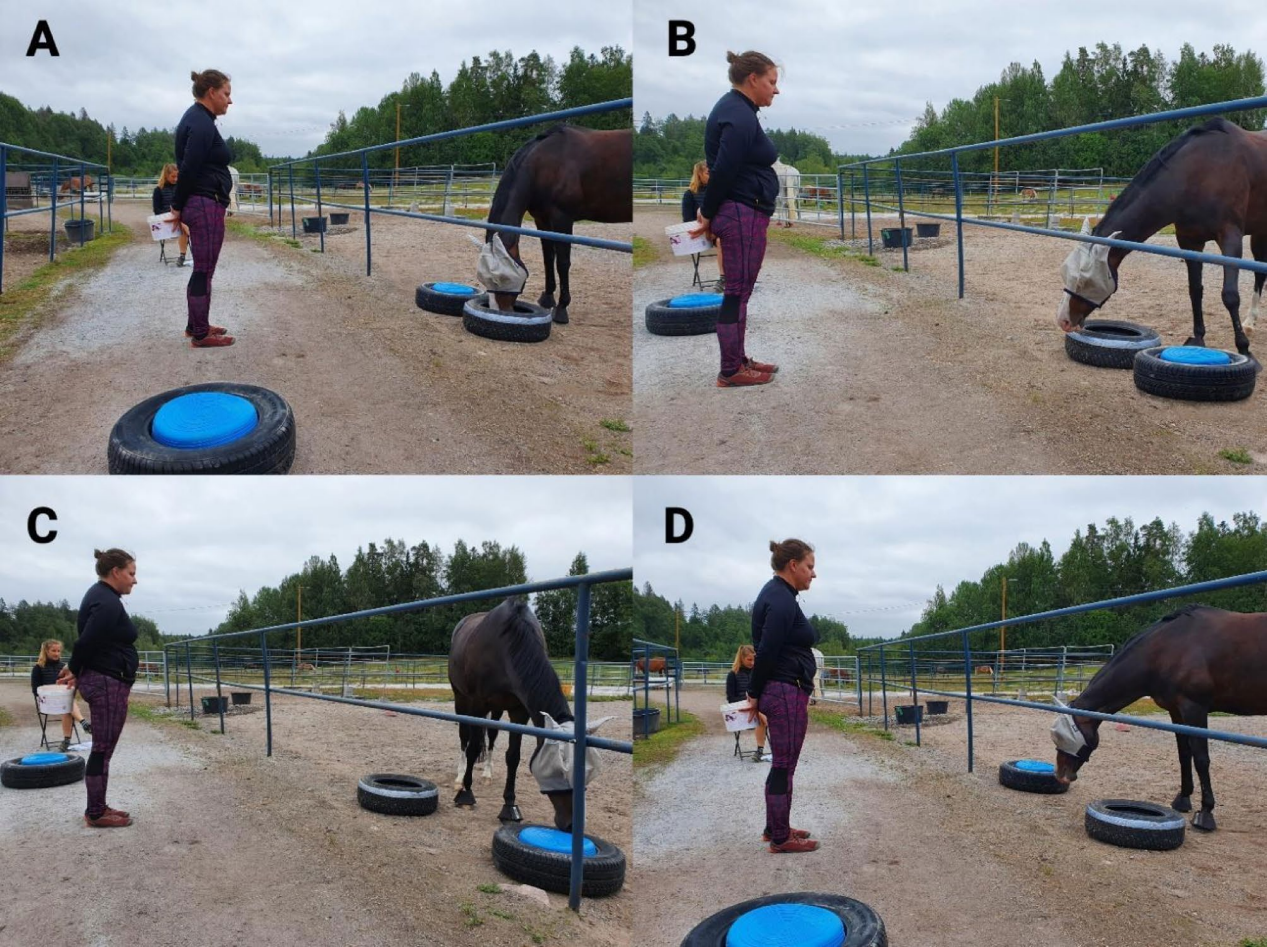
Setup of the preschooling phase of the reversal learning test: Horses were gradually introduced to the test objects by presenting them one at a time in the following order: (**A**) Object 1 placed near the bucket, (**B**) Object 2 placed near the bucket, (**C**) Object 2 placed far from the bucket, and (**D**) Object 1 placed far from the bucket.

Preschooling was based on operant conditioning methodology and consisted of four phases in which touching the object was generalized to different locations (Fig. 3). Each phase continued until the horses made four fluent repetitions. Fluent repetition was subjectively assessed by Experimenter 1 and was defined as having a latency of 1–2 s at most, with no other behaviors present. In practice, this meant that the horse was eating the treat and then went straight to touch the object again. The horse was allowed to look around while chewing, with this exception. If a horse touched the object several times but was not going to eat the treat, every touch was reinforced but only eating was counted as a correct response. Both Experimenters (1 and 2) counted the repetitions. Between the repetitions, Experimenter 1 was standing in front of the feed bucket, 2 m away from the fence, shoulder line facing the feed bucket and eyes following the horse’s muzzle, holding a small bucket filled with treats behind their back (Fig. 3). After both objects were presented in all four locations, repetitions were balanced to ensure that each object and side were reinforced equally, preventing any later bias toward one object.

### Reversal learning test (RLT)

During the preschooling phase, horses were trained to follow the object location (i.e. left or right) for reinforcement. The RLT started with a memory test performed in the same way as preschooling ended. The right object was the last reinforced in the memory test (Supplementary Fig. 1; phases 8–9).

**Fig. 4:**
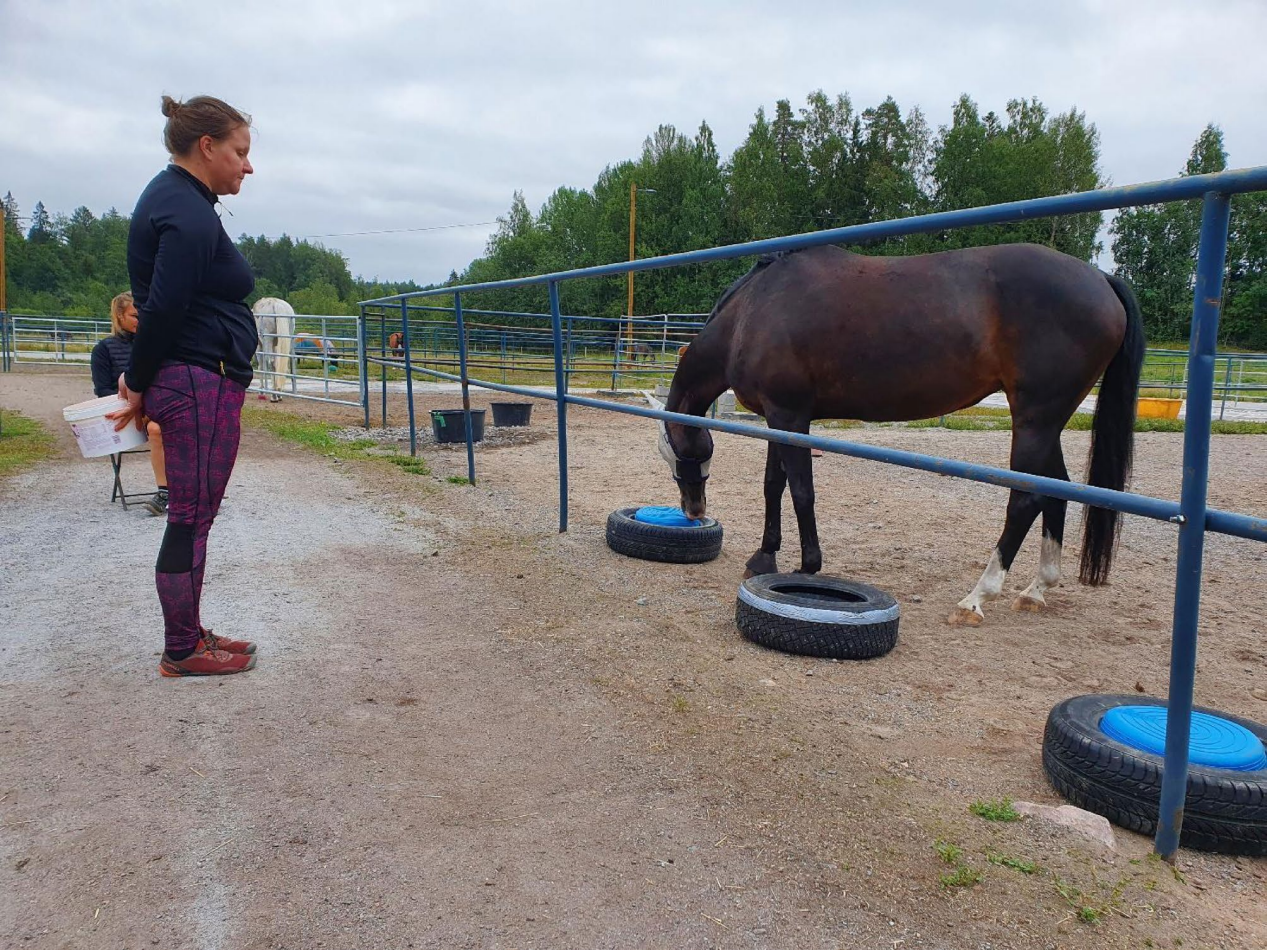
Setup of the reversal learning test.

Experimenter 1 placed a few carrot slices in the feed bucket, and then both objects were simultaneously placed into the training area (Fig. 4). The horses were allowed to freely choose their starting side. Experimenter 2 recorded the time from the first repetition and counted the correct touches per minute.

Based on pilot observations, the learning criterion was set at seven correct responses per minute. This threshold was established after testing six horses to identify the point at which extraneous behaviors ceased, at which point all of those horses achieved at least seven correct responses. The criterion was selected to be attainable for horses of different sizes and temperaments once the behavior became fluent. When the horse reached the criterion, the correct location (i.e., left or right) was reversed. To prevent horses from associating specific words (e.g., ‘reverse’) with the task, Experimenter 2 spoke random words once or twice per minute. When a word from a predetermined category (e.g., flowers, domestic animals) was used, it signaled Experimenter 1 to initiate a reversal. Different words from the same category were used for each reversal to avoid any unintentional learning by the horse. The RLT continued for up to 30 min, until one kilogram of carrots had been eaten, or if the horse left the training area and showed no engagement with the task for more than 2 min.

When reviewing the results later, an 80% success rate was established as the learning criterion and a sign of completing the test. Previously, a minimum of 80% correct responses has been used as the threshold for learning^48,51^, although the rationale for this is rarely explained. This threshold was chosen because Wilson (2019) suggested an optimal training accuracy of about 85%^59^, and Buechel et al. (2018) found no significant improvement in performance above an 80% success rate in a RLT^60^.

### Behavioral recordings

The preschooling and actual RLT testing situations were recorded with a small camera (GoPro Hero 7 Black, China, 2018) placed two meters from the testing site. From the videos, we counted errors and behavioral indicators of acute frustration per minute. Errors were defined as the horse’s muzzle touching the wrong object, with the first incorrect touch after a reversal not counted as an error. Frustration behaviors were recorded on a yes/no basis for each minute of observation and included increased locomotion (e.g., pawing, tapping, or waving a foreleg), direct aggression toward equipment (e.g., biting, pushing, or knocking objects over with the muzzle), and conflict or displacement behaviors (e.g., wind-sucking, self-scratching)^61^.

Nighttime behaviors of the horses were recorded as part of a separate experiment using video cameras (Zhejiang Dahua Technology, China, 2019). The cameras were positioned above the stalls to simultaneously film two horses. Recordings were conducted over two consecutive nights from 4 PM to 8 AM, starting with the baseline at week 0. Subsequent recordings took place during weeks 1, 3, 4, and 6 of the bedding experiment. The RLT was conducted during week 6, at least two days prior to the final video recording. Scoring was performed continuously using a predefined ethogram for head, neck, and body postures; REM-like sleep was scored when the horse was lying down on its side or sternum with a relaxed neck that did not support the head^14,62^. The videos were scored in a randomized order using CowLog software^54^ by a single rater who was blind to the results of the learning test and the depth of bedding.

### Statistical analysis

Prior to statistical analyses, the mean nighttime REM-like sleep duration of the horses was categorized as either < 30 min or ≥ 30 min. Horses were also classified as ‘quitters’ (those that voluntarily ended the RLT) or ‘motivated’ (those that consumed all rewards or completed 30 minutes of test duration). The time lag between consecutive reversals, the number of errors per reversal, and the minutes with frustration behaviors per reversal were recorded. Overall parameters were calculated per reversal and/or per minute, and the occurrence of reversals per minute and frustration behaviors per reversal were coded as binary variables (yes/no). The dataset included the first 15 reversals while three horses were still participating.

The overall data were analyzed using generalized linear models (GENLIN) with a normal distribution, identity link, and maximum likelihood (MLE) estimation. The models examined the effects of mean REM-like sleep duration on: (1) motivation (quitters vs. motivated) and (2) the mean number of reversals. Additional models tested the effect of motivation on: (3) mean REM-like sleep duration and (4) the mean number of reversals.

Continuous longitudinal variables were analyzed using linear mixed models that accounted for repeated measures. These models examined differences between reversals in (1) the number of minutes with frustration behaviors prior to each reversal and (2) the proportion of errors during reversals. Reversals were included as a repeated fixed factor, with horse as a random factor, and an autoregressive (AR(1)) covariance structure was applied to account for within-subject correlations. REM-like sleep was excluded from the final models due to non-significance. Residuals were inspected graphically to confirm normality.

Binary repeated measures (i.e., the occurrence of frustration behaviors and reversals) were analyzed using generalized estimating equation (GEE) models with a binomial distribution and logit link function, along with an AR(1) working correlation matrix. The effects of REM-like sleep duration (treated as a continuous variable) on: (1) the occurrence of reversals and (2) frustration behaviors were evaluated. Odds ratios (OR) and 95% Wald confidence intervals were obtained by exponentiating the model coefficients.

Additionally, we tested for differences in learning curves between categorized REM-like sleep variables using a Kaplan-Meier test. Statistical significance was set at *p* < 0.05.

## Results

### Preschooling phase

All horses tested were able to reach the target training task. The (mean ± SE) duration for preschooling phase was 18 ± 2 min varying between 7 and 41 min. Three of the horses needed one and one horse needed three breaks during the preschooling due to environmental disturbances. Horses required an average of 23 ± 2 repetitions per object to reach fluent behavior, with a range of 12 to 43 repetitions. The required number of repetitions was then adjusted so that each horse had the same number of repetitions for each object, resulting in a mean total of 51 ± 4 repetitions. One kilo of carrots was a working amount, and horses needed 457 ± 29 g on average to learn the task.

### *RLT*

Ten horses began the RLT with the right object, while six started with the left object. All horses could reach the predefined rate of seven correct touches per minute. Of the horses, 15 out of 16 were able to reverse three or more times and completed the RLT with a mean ± SE number of 9 ± 1 successful reversals in 22.2 ± 1.4 min and those horses did 114 ± 6 correct touches during the RLT. One horse did not understand the concept of reversal learning and quit the test after the second reversal, when behavior towards both objects was extinguished. Carrots were the limiting factor in the RLT for nine horses, and six horses quit the test. One horse still had rewards remaining and the motivation to continue for more than 30 min. The quitters achieved a mean of 6 ± 2 reversals, CI 95% [3, 9], which differed from the motivated horses, who achieved a mean of 11 ± 1, [8, 13] (*p* = 0.03).

**Fig. 5:**
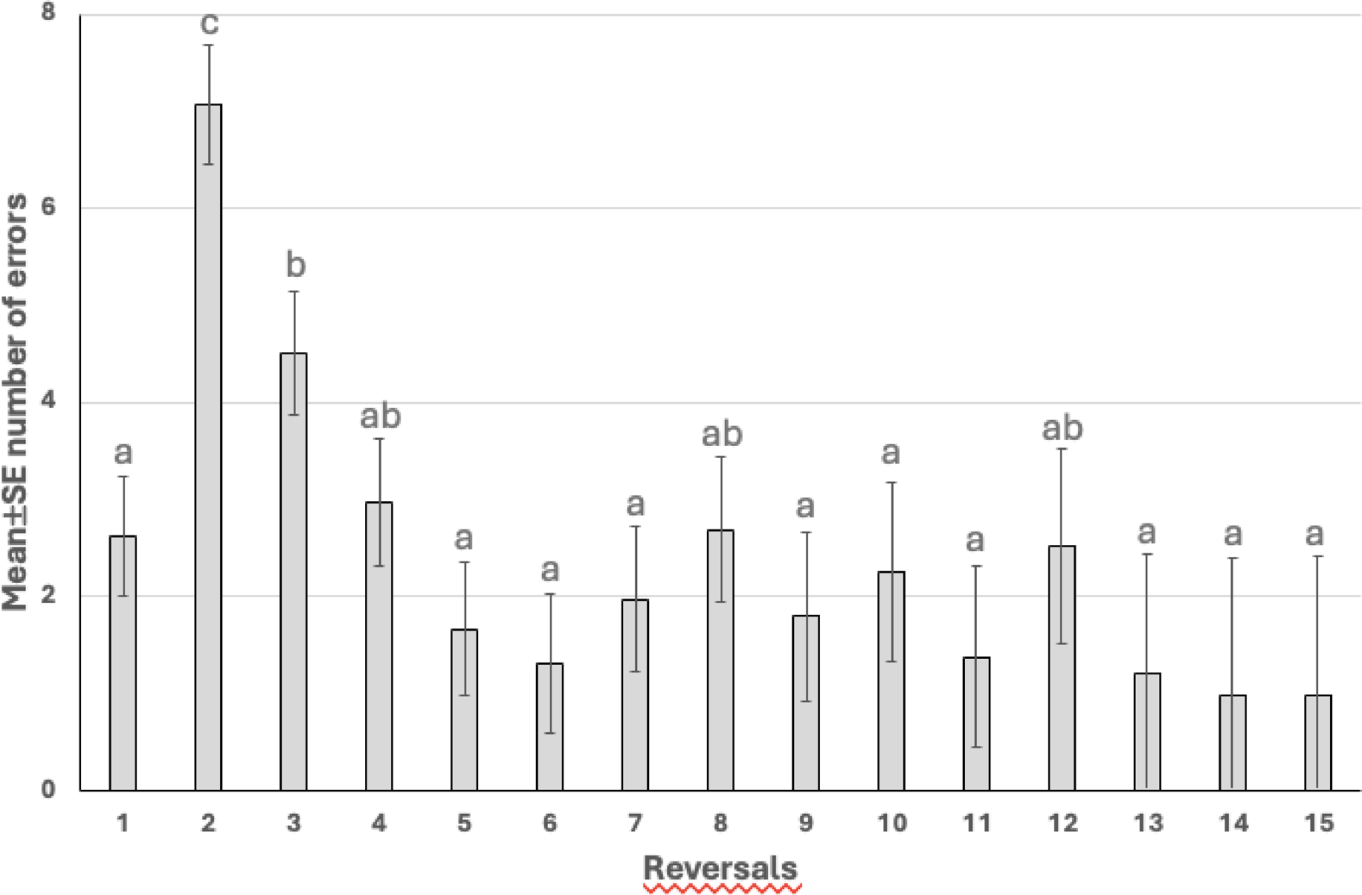
Mean ± SE number of errors before reversal in horses performing a reversal learning test. Different letters between reversals indicate the statistically significant difference (*p* < 0.05).

**Fig. 6:**
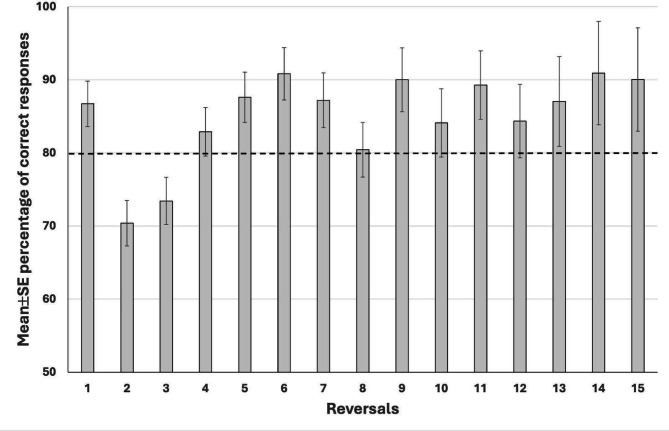
Mean ± SE percentage of correct responses in horses (*n* = 15) performing a reversal learning test. Dashed line marks 80%.

Overall, the horses had 2.4 ± 0.4 errors per reversal, and the mean proportion of correct responses was 85.0 ± 1.6% (range 67.7–93.4%). However, these were affected by the progress of the test (*p* < 0.001 for both). Horses made most of the errors before the second and third reversal. After the third reversal errors returned to the initial level (Fig. 5). In addition, the mean correct response was the lowest during the 2nd and 3rd reversals, after it reached a level of greater than 80% (Fig. 6).

The mean time lag between reversals was 1.9 ± 0.2 min, and it was affected by the progress of the test (*p* = 0.003), with the 2nd reversal being the longest at 4.3 ± 0.4 min, differing significantly from all subsequent reversals (*p* < 0.05 for all).

### REM-like sleep

The overall mean ± SE REM-like sleep duration the horses exhibited during the observation period was 46.1 ± 8.7 min. However, ten horses exhibited REM-like sleep less than 30 min (10.6 ± 2.2 min, range 0.0–28.0 min), while six exhibited REM-like sleep for at least 30 min (42.3 ± 4.8 min, range 36.6–65.8 min). The overall amount of REM-like sleep differed between quitters and motivated horses: 24.2 ± 11.9 min, Cl 95% [0.8, 47.6] min vs. 59.3 ± 9.24 min, [41.2, 77.4] min, respectively (*p* = 0.02).

The occurrence of reversals during test minutes was not significantly affected by either test progress or mean REM-like sleep duration (*p* > 0.05 for both). However, the overall amount of REM-like sleep was positively associated with the total number of correct reversals during the RLT (B = 0.06, SE = 0.03, CI 95% [0.01, 0.12], *p* = 0.04). In contrast, it was not significantly associated with the proportion of errors among all reversals (B=−0.06, SE = 0.05, CI 95% [−0.15, 0.03], *p* > 0.05).

**Fig. 7:**
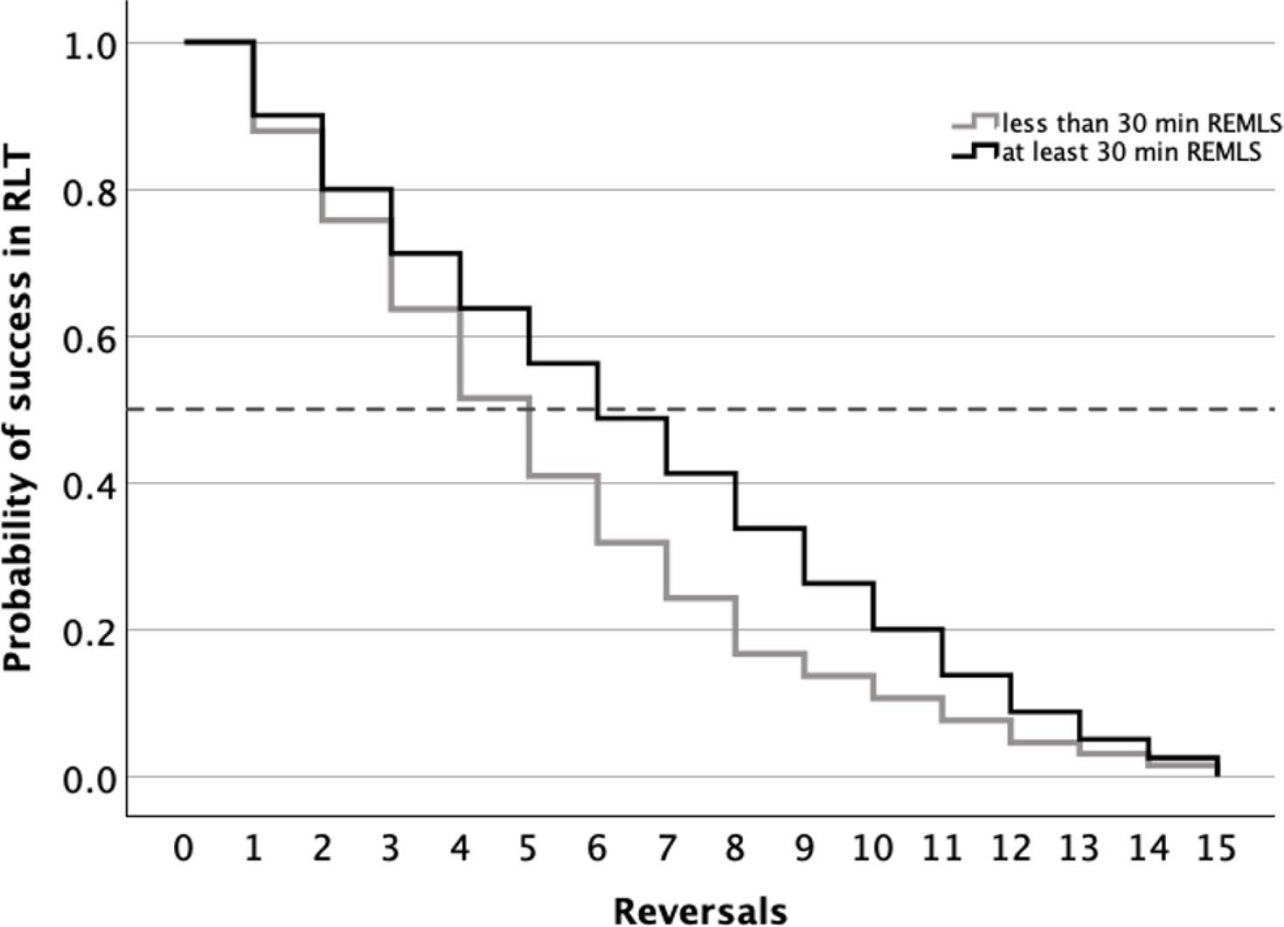
Kaplan-Meier survival curve in reversal learning test (RLT) comparing 15 horses with mean duration of night-time rapid-eye-movement-like sleep behavior (REMLS) measured from 8 nights over the previous 6 weeks period. Duration was scored as less than 30 min (*n* = 10) and at least 30 min of REMSL (*n* = 6).

In addition, a log-rank test showed differences between short and long REM-like sleep in the probability of moving forward on the test (*p* = 0.049); a 50% chance of a horse moving forward in the test occurring in a median [CI 95%] of 5 [4.0, 6.0] reversals for horses with less REM-like sleep and in 6 reversals [4.5, 7.5] for horses with more REM-like sleep (Fig. 7).

### Frustration behaviors

**Fig. 8:**
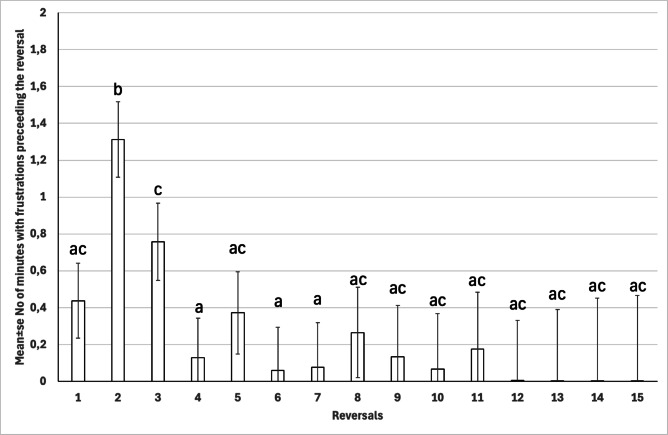
Mean ± SE number of minutes with frustration behaviors preceding reversal in horses (*n* = 15) during the reversal test. Different letters between reversals indicate the statistically significant difference (*p* < 0.05).

Frustration behaviors were observed in 14 out of 16 horses during the RLT. The horses exhibited frustration during 16.5 ± 4.7% of all RLT test minutes. The overall number of minutes with frustration behaviors preceding one reversal was 0.3 ± 0.1 min. The number of minutes with frustration behaviors preceding a successful reversal was significantly affected by the progress of the test (*p* = 0.002). Frustration peaked during the second reversal and gradually decreased thereafter, returning to baseline levels as the test progressed (Fig. 8).

The overall proportions of minutes with frustration behaviors were not statistically different between quitters and motivated horses (9.2 ± 2.9%, Cl 95% [3.5, 14.9] vs. 10.1 ± 2.3%, [5.7, 14.5], (*p* = 0.81). However, in the GEE models, the likelihood of frustration behaviors during the test decreased significantly as the task progressed. Additionally, each additional minute of mean REM-like sleep duration was associated with a lower probability of frustration behaviors (*p* < 0.05 for both, see Supplementary Material 2).

## Discussion

We show here a method to perform a practical reversal learning test in a relatively short time for a group of horses in their home premises. To our knowledge this is the first report of a simple, frequency-based, spatial reversal learning test for horses. In accordance with the hypothesis, we found that horses with shorter REM-like sleep durations performed worse on the RLT than those with longer durations. Horses that exhibited more REM-like sleep worked significantly longer than those with less REM-like sleep behavior, suggesting that this reflects their perseverance or motivation rather than their ability to understand and apply the concept of reversal learning.

Despite differences in the duration of REM-like sleep, all horses except one performed successfully in the RLT and did not show clear differences in their cognitive flexibility^29^. However, we also found that horses that quitted the RLT themselves, before the end of the rewards or test time, had less REM-like sleep than those that continued until the end of the test or rewards. This is in line with the findings with various other species showing that reduced REM sleep is associated with suboptimal mood, performance deficits, and impaired learning^11,63–66^. Our research horses were not subjected to artificial sleep deprivation, and their sleep was measured over several nights in their home environment to determine their sleep phenotype. None of the horses exhibited clinical signs of REM sleep deprivation^25^.

We observed that short REM sleep durations affected performance. This is interesting because, although horses as a species have the evolutionary ability to adapt to suboptimal conditions by reducing their REM sleep^12^, this adaptation does not appear to come without a cost. Despite reduced REM-like sleep, the horses were able to learn simple tasks, such as target work. However, when the task was prolonged, their motivation to continue the test seemed to decline. Research in humans has shown that sleep deprivation does not necessarily affect initial test progress, as increased alertness and strong motivation can mask fatigue; however, differences begin to emerge when the test is prolonged^26^. Further research is warranted to understand how horses with clinical signs of REM sleep disturbance^25^ solve reversal learning problems.

The developed RLT presented here was feasible in field conditions and meets the criteria of a practical learning test. All horses were able to reach the pre-set criterion of correct touches per minute. Our results suggest that measuring the frequency of correct responses per minute is a practical and sensitive way to monitor horses’ learning progress without interrupting training. Traditionally, learning is measured by reduced latency and errors^36,46^; however, in long training sessions, this can be difficult to assess accurately. Counting the frequency of successful responses offers a practical alternative to mean error rates or fixed success thresholds^60^. Our approach differed from previous studies, where learning was defined as eight consecutive correct responses with tasks presented every other day^36,38^. We observed that horses began to anticipate the reversal after a certain number of consecutive correct touches, suggesting that such designs may measure task chaining rather than true reversal learning, reflecting the horses’ ability to sequence learned behaviors^67,68^. However, this should be studied further in horses.

As expected, correct responses and errors per reversal decreased as the RLT progressed and the horses learned the concept. Consistently with previous studies^37–39^ horses made the majority of errors prior to the second reversal, after which error rates returned to initial levels by the third reversal. Accordingly, correct response rates were lowest before the second reversal but rose to 80% or higher following the third reversal. In our study, horses learned the initial discrimination during preschooling, unlike in Warren & Warren^36^, where two targets were used from the start and classical conditioning, rather than operant conditioning, was applied. As Harlow^33^ noted, previously acquired discrimination learning facilitates transfer to reversal learning tasks. The error curve observed by Warren & Warren closely matched our results; their horses also averaged about two errors per problem once they understood the reversal concept.

Almost all horses showed signs of frustration during the RLT, but this frustration decreased with each reversal as they learned the task concept. Frustration behaviors were also associated with REM sleep duration; longer REM sleep was linked to a reduced risk of exhibiting frustration. This aligns with Skinner’s^69^ description of extinction, where frustration arises during learning but gradually leads to behavioral adaptation. We observed a wide range of frustration behaviors directed at the objects, such as pawing, biting, and pushing with the muzzle, which may reflect individual temperament and coping styles. We also noted self-grooming, and one horse displayed stereotypic behavior (wind-sucking), further emphasizing how behavioral responses in the test can reveal aspects of the horse’s personality and emotional regulation. In the future, the RLT could also be used to assess how individual horses cope with challenging situations and express frustration, similar to how temperament and behavior tests are used in dogs^70^.

In our experience, several factors should be considered when designing and conducting an RLT test on horses under field conditions. To minimise stress and reduce the influence of the horses’ prior learning, experiences, and surroundings, horses should be tested in its familiar paddock, without being led or in close contact with humans at any point. Research shows that horses perform best in familiar environments^71,72^. To avoid measuring the horse’s prior experiences in the RLT rather than the ability to adapt to new learning challenges, preschooling was crucial. Four fluent repetitions were sufficient to confirm learning, and a memory test—administered on the same day just prior to the RLT—validated that the task had been learned and ensured that the horse still understood it.

Horses were willing to work over a hundred repetitions during the RLT. In earlier studies learning sets were often short and repeated over several days^36,38,39,46,48,51^. We argue that long training sessions are not problematic if the teaching is logical for the animal, progresses in small steps, and minimizes the possibility of errors, with timely rewards for clearly understood tasks. We chose a spatial test because horses learn left/right cues better than visual ones^39^. We placed the targets 90 cm apart to ensure that horses had a clear choice between them while keeping the distances short enough to maintain their focus. In real life, humans can demand long-lasting, sometimes unclear, and repetitive tasks from horses, which can hinder performance, increase unnecessary stress, and exacerbate sleep disorders.

This study has some limitations. The RLT was conducted on riding horses in their natural environment. Although the setting was familiar to them, several environmental and physiological factors that may have influenced the results could not be standardized. The small sample size may have limited the detection of some associations between sleep and the measured parameters. For example, undiagnosed health issues could have affected the horses’ motivation to touch the objects, while unexpected disturbances in the environment might have interfered with test performance. Both factors could also have influenced sleep patterns, potentially confounding the observed relationships between sleep and test performance. Furthermore, it is possible that, even in the absence of sleep disturbances, some horses are inherently less persistent in tasks requiring sustained cognitive control over an extended period^73,74^. In this study, all horses were given a piece of carrot as a reward, which worked well; however, individual differences in preferences or chewing ability may have affected motivation.

The complete test requires only two people, a maximum of two hours of time per horse, two kilograms of carrot slices, and two targets, making it easy to implement in field conditions. For human safety, the subject and the horse were separated by a fence, but the human remained visible during the exercise. This setup presents a risk of the Clever Hans effect^52^, where the horse may unconsciously learn from human cues, such as body language or spoken words^71^. However, the visual barrier would also introduce a new element for the horse to adapt to. Based on our data, where all horses touched the wrong object at least once after the reversal, the Clever Hans phenomenon appears to have been avoided. However, it is possible that this could become an issue in future repetitions of the test. In developing this test, we observed that horses can quickly assimilate and learn words, which justifies the random selection and timing of the words used.

The test requires the experimenter to show basic animal training skills. Two-thirds of the test developers were trained animal trainers. The timing of the secondary reinforcer by the experimenter is crucial for successful testing; if it is delayed, there is a risk of unintentional behaviors, such as pawing or fussing with the objects. Another challenge for an inexperienced trainer may be determining fluent repetition next to the horse. Fluency is naturally subjective, even if it is precisely defined. Although it remains consistent throughout the preschooling phase when the same experimenter is involved.

We concluded that the reversal learning test (RLT) can be conducted in the horses’ natural environment, as they are turned out in a paddock. While more studies are needed, differences in RLT performance appear to be linked to the amount of REM-like sleep. If there are concerns about a horse’s learning ability, it would be beneficial to assess it using the RLT. We observe differences in how horses perform in the RLT, but we have yet to uncover all the possible causes for these differences. The RLT situation seems to mimic real-life challenges effectively.

## Supplementary Information

Below is the link to the electronic supplementary material.

Supplementary Material 1

Supplementary Material 2

## Data Availability

All data supporting the findings of this study are publicly available in the Zenodo repository at [**https://doi.org/10.5281/zenodo.17979374**](https:/doi.org/10.5281/zenodo.17979374).
