# Supplementary figures and images for: Effect of horse sleep behavior on performance in a field-side spatial reversal learning test
Source: Sci Rep. 2026 Jan 6;16:4265. doi: 10.1038/s41598-025-34463-9 (PMC12858803; doi:10.1038/s41598-025-34463-9)

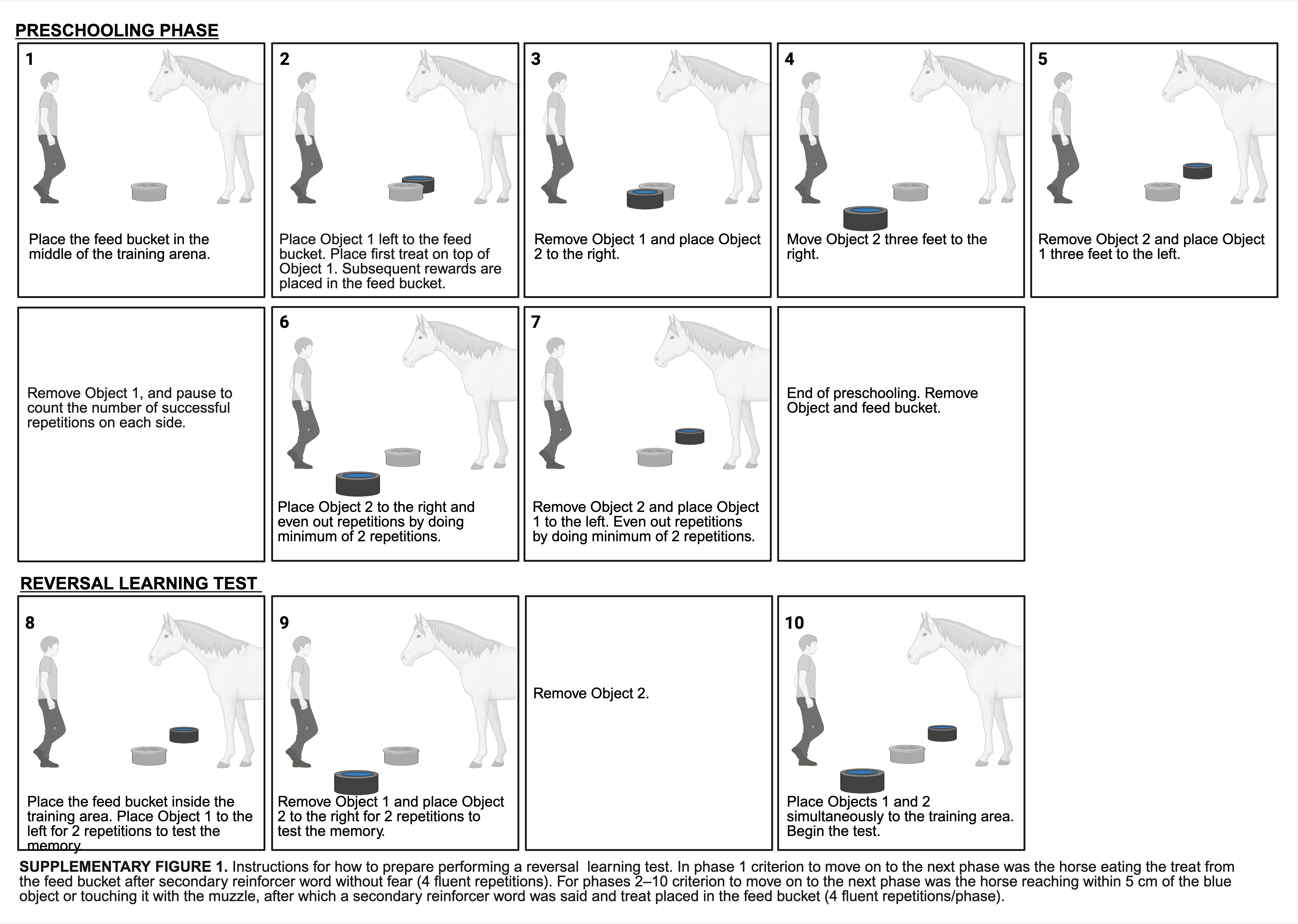

Supplement: Supplementary file 1 — Supplementary Material 1 [file 41598_2025_34463_MOESM1_ESM.jpeg]
