# Supplementary material for: Effect of horse sleep behavior on performance in a field-side spatial reversal learning test
Source: Sci Rep. 2026 Jan 6;16:4265. doi: 10.1038/s41598-025-34463-9 (PMC12858803; doi:10.1038/s41598-025-34463-9)
Supplement: Supplementary file 2 — Supplementary Material 2 [file 41598_2025_34463_MOESM2_ESM.docx]

Supplementary Table 1. Effects of test progress (in minutes) and the 6-week mean nighttime REM-like sleep (REMLS) duration of horses (n=15) on frustration behaviors and reversal occurrences during reversal learning test minutes, based on generalized estimating equation (GEE) models (odds ratios with 95% confidence intervals and p-values).

| **Dependent** | **Parameter** | **OR [CI 95%]** | **p** |
| --- | --- | --- | --- |
| Occurrence of frustration behaviors during test minute | (Intercept) | 1.89 [0.54, 6.57] | 0.32 |
|  | Progress of the test (min) | 0.80 [0.74, 0.87] | 0.001 |
|  | Mean REMSL duration (min) | 0.98 [0.96, 1.00] | 0.04 |
